# Innate immune pathway activation to modulate mesenchymal stromal cell (MSC) interactions with synovium and cartilage

**DOI:** 10.3389/fbioe.2025.1605148

**Published:** 2025-08-08

**Authors:** Peter Linde, Lyndah Chow, Isabella Sabino, Zoë Williams, Renata Impastato, Steven Dow, Lynn Pezzanite

**Affiliations:** Department of Clinical Sciences, College of Veterinary Medicine and Biomedical Sciences, Colorado State University, Fort Collins, CO, United States

**Keywords:** osteoarthritis, mesenchymal stromal cell, regenerative therapies, STING, toll-like receptor, pattern recognition receptor

## Abstract

**Introduction:**

Mesenchymal stromal cells (MSCs) have been evaluated as a local therapeutic option to treat osteoarthritis (OA) with conflicting clinical results. Our previous studies have evaluated immune licensing of MSC through activation of Toll-like receptor and cytosolic cGAS-STING pathways, with demonstrated improvement in functional and structural outcomes in a rodent model of OA. The objective of this study was to investigate impact of MSC activation on their interaction with relevant joint target cells to better understand the mechanisms by which pre-activation improves MSC activity for treatment of osteoarthritis.

**Methods:**

Equine bone-marrow-derived MSCs (passage 2–3) from 3 healthy donors were stimulated with a TLR3-pathway agonist (polyinosinic:polycytidylic acid) or STING pathway agonist (2′3′-cGAMP) (10 μg/mL, 2 h, 2 × 10^6^ cells/mL in suspension). Cells were plated (100,000 cells/well, 24-well plates) and conditioned media (CM) collected at 24 h. Equine monocyte-derived macrophages, synovial cells, and chondrocytes were stimulated with IL-1ß/TNF-α (20 ng/mL, 2 h), washed and treated 24 h with MSC-CM, TLR-MSC-CM or STING-MSC-CM, washed and cultured 24 h. CM was examined for cytokine secretion by multiplex immunoassay and ELISA (25 cytokines). Bulk RNA sequencing was performed on MSC and joint cell lines via an Illumina based platform.

**Results:**

TLR-MSC-CM decreased IL-1β (p = 0.02), IL-6 (p = 0.02) secretion by synoviocytes and IL-18 secretion by activated chondrocytes (p = 0.002). STING-MSC-CM decreased IL-6, IL-8 secretion (p = 0.08) by synoviocytes, decreased IL-8 (p = 0.05) by activated chondrocytes, increased G-CSF (p = 0.01), IL-4 (p = 0.01) and decreased IL-5 (p = 0.01) by activated macrophages. Transcriptomic analyses indicated differential gene expression in each cell line following CM treatment varied by cell line. STING-MSC-CM vs TLR-MSC-CM induced 38 significantly altered DEGs in synoviocytes, 20 in chondrocytes, and 47 in macrophages.

**Discussion:**

These findings indicate that joint cells respond differently to factors secreted by TLR or STING pathway activated MSC. The pathways altered were different for each target cell type and no clear pattern of responses was apparent. These results indicate that *in vitro* modeling of target cell responses to “licensed” MSC can provide new information on the MSC and target cell interactions, though ultimately the functional impacts of activated MSC need to be evaluated using *in vivo* models.

## Introduction

Osteoarthritis (OA) is a common degenerative joint disease in companion animal species and humans, resulting in pain and socio-economic burden. Estimated to affect up to 80% of horses over 15 years old, and one-third of people over 65, OA is the second most costly health condition treated in the US (BMUS, 2024). The comparable disease prevalence and rate of progression, articular cartilage loading forces, cartilage thickness and joint volume between horses and humans, and greater ease with which to obtain equine synovial tissues for modeling makes studying spontaneously occurring OA using horse tissues *in vitro* a valuable translational model for both species (Frisbie et al., 2006; Chu et al., 2010; Wayne Mcilwraith et al., 2011; Reesink et al., 2017). Despite the high frequency and economic burden of OA across species, there are no approved interventions or treatments to mitigate or reverse joint degeneration.

Regenerative therapies, including mesenchymal stromal cells (MSCs), have gained increasing recognition for their therapeutic and immunomodulatory potential in OA but have reportedly variable efficacy which has been attributed potentially in part to heterogeneity between donors and within culture populations. Priming MSCs with immune stimulants that activate pattern recognition receptors (PRRs) on their surface such as TLR-3 agonist polyinosinic:polycytidylic acid (poly I:C) may increase homogeneity and the clinical effectiveness of cell products and therefore ultimately treatment efficacy (Krampera, 2011; Szabó et al., 2015; Johnson et al., 2017; Cassano et al., 2018; Pezzanite L. M. et al., 2021; 2022). We have recently shown pre-activation of MSC with two PRRs, Toll-like-receptor (TLR)-3 polyinosinic:polycytidylic acid (pIC) and the STimulator of Interferon Genes (STING) pathway agonist 2′,3′-cGAMP improves histologic and functional gait outcomes in a murine model of OA (Plaisance et al., 2025). Activation of STING pathways has been previously described to induce production of Type I interferons in immune and sensory cells following tissue injury, with demonstrated potential to be both inflammatory and antinociceptive depending on the context in which it is injected (Liu et al., 2016; Donnelly et al., 2021). While STING pathway activation has been evaluated in the context of nociception and regulation of neuropathic pain, our recent work represents the first use of STING pathway agonists to stimulate MSC to induce an immunomodulatory response in the context of OA (Ishikawa and Barber, 2008; Donnelly et al., 2021; Wang et al., 2021; Sun et al., 2022).

Once thought to result exclusively from biomechanical forces causing cartilage injury and subsequently propagating erosive changes, OA is increasingly recognized to be an immunologically mediated disease, wherein both local and systemic immune responses drive destruction of joint tissues. Current understanding of OA pathogenesis supports a key role to innate immune effector cells for regulating and perpetuating low-grade inflammation associated with OA, which may be modulated by treatment with MSC therapy. Macrophages are the most numerous immune cell type in the synovium and play a key role in sustaining inflammation through release of inflammatory mediators (*e.g.*, cytokines, MMPs, ROS/RNI metabolites) in response to damage-associated molecular patterns released in the joint (Fahy et al., 2014; Fichadiya et al., 2016; Manferdini et al., 2016). We hypothesized that the observed beneficial effect of injecting STING pathway activated MSC could include suppression of inflammatory pathways and upregulation of anabolic pathways in joint target cells, including chondrocytes, synoviocytes, and macrophages. We sought therefore to investigate the impacts of activated MSC on these target cells, using *in vitro* assays with target cells and MSC CM.

The objective of this study was to use *in vitro* bioassays to compare the anti-inflammatory and immunomodulatory properties of TLR3 pathway- or STING pathway-activated MSC when secreted factors from these cells interact with synoviocytes, chondrocytes and macrophages. The assays used CM from activated equine bone-marrow derived MSC and cultures of equine chondrocytes, synoviocytes and macrophages from healthy animals to assess the impact on target cell cytokine production and transcriptomic responses. Our analyses revealed induction of interferon related pathways in target cells exposed to either TLR3-or STING-pathway activated MSC, and variable changes in cytokine production. These findings indicate that ‘immune licensing’ of MSC prior to *in vivo* injection in OA can alter joint target cell responses in multiple different ways, including modulation of immune pathways. Overall, this work provides new insights into how immune-licensed MSC interact with joint cells to modulate local inflammation and disease progression.

## Methods

### Study overview

**FIGURE 1:**
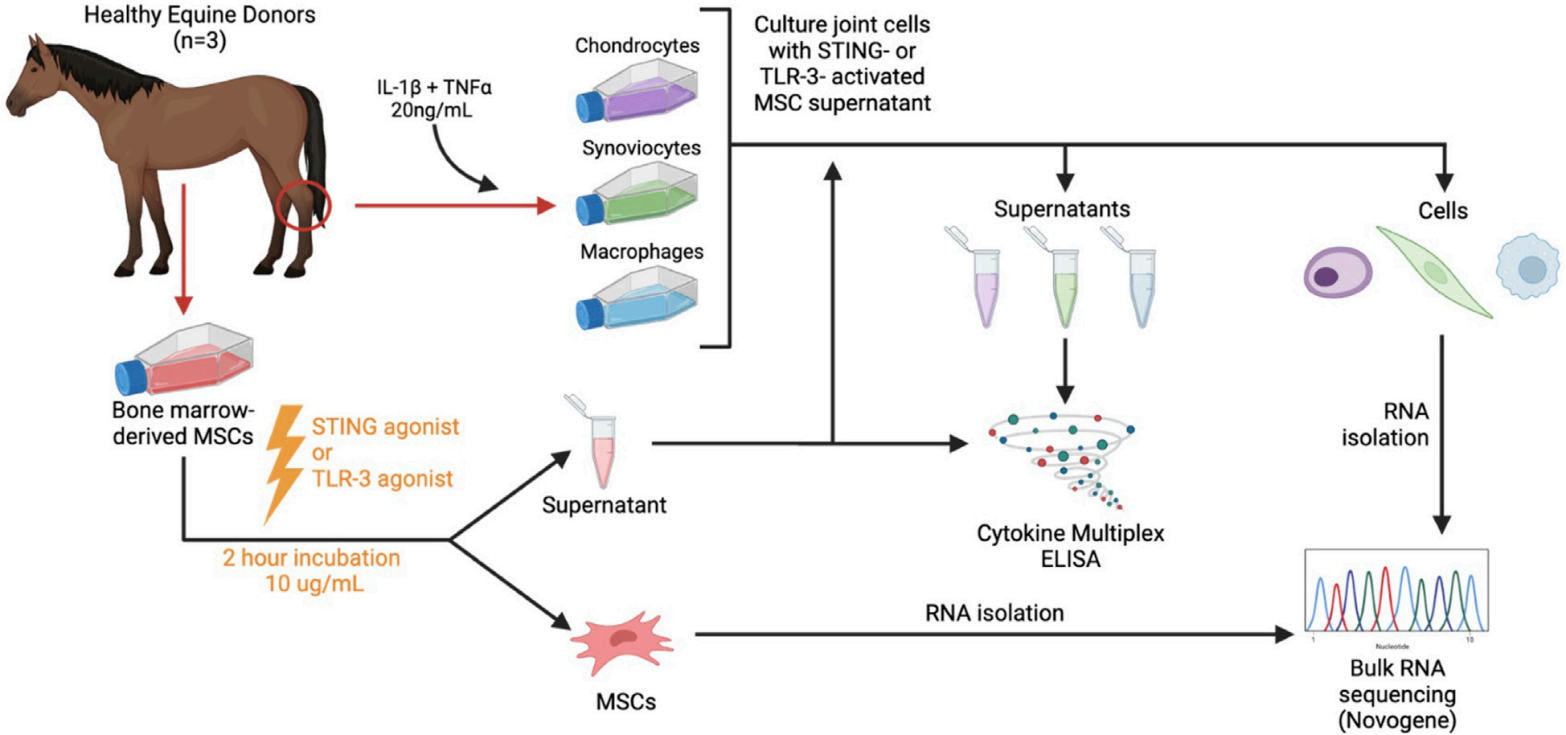
Study overview. Horses (n = 3 total) were donors of bone marrow aspirate for mesenchymal stromal cell culture (MSC), which were activated with either a TLR3 agonist (poly I:C) or STING agonist (2′3′-cGAMP) and conditioned media were generated. Activated or non-activated conditioned media were then applied to key joint cells (synoviocytes, chondrocytes, macrophages) and cells were assessed for relative cytokine secretion and transcriptomic expression following application.

The Institutional Animal Care and Use Committee at Colorado State University (No. CSU IACUC #5672) approved tissue collection for this study. All methods were conducted according to the national guidelines under which the institution operates and NIH Guidelines for the Care and Use of Laboratory Animals (8^th^ edition). Horses (n = 3 total) were donors of bone marrow aspirate for mesenchymal stromal cell culture, which were activated with either poly I:C or 2′3′cGAMP, conditioned media were generated, and cells were submitted for transcriptomic analyses to determine gene expression following activation, as described below. Activated or non-activated conditioned media were then applied to key joint cells (synoviocytes, chondrocytes, macrophages) from one healthy donor horse and cells were assessed for relative cytokine secretion and transcriptomic expression. An overview of the experimental workflow is shown in Figure 1.

### Mesenchymal stromal cell culture

MSCs were isolated from bone marrow aspirates collected from the sternum of donor horses (n = 3, Quarter Horses, 3–4 years old, 2 females and 1 male) as previously described (Pezzanite L. et al., 2021). MSCs used for *in vitro* studies are routinely evaluated for surface phenotype and found to be CD44^+^CD90^+^ and CD34^−^CD45^−^using equine cross-reactive antibodies as previously described (Esteves, et al., 2017) and in accordance with minimal criteria to define MSCs as described by the International Society for Cellular Therapy (Dominici et al., 2006). To generate MSC conditioned supernatant, cells were thawed in a 37°C water bath and recovered overnight. Then they were plated on 24 well-plates at 100,000 cells/well cultured 24 h in complete growth media, activated with poly I:C (10 μg/mL, Invivogen) or 2′3′cGAMP (10 μg/mL, Invivogen) for 2 h and subsequently washed with PBS. Complete growth media was added and after 24 more hours, conditioned supernatants were collected and frozen in −20°C, and cells were lysed and frozen in RLT lysis buffer (QIAGEN) for RNA analysis.

### Synovial cell isolation and treatment with conditioned media

Synoviocytes were obtained from synovium of one horse; briefly, synovium was excised from the femoropatellar joint, minced and digested for 4 h in complete media (Dulbecco’s Modified Eagle Medium, 10% FBS, penicillin (100 units/mL), streptomycin (100 ug/mL), 1 M HEPES) with type II collagenase (0.75 mg/mL), as previously described (Bolt et al., 2008; Pezzanite L. M. et al., 2021). Digested synovium was filtered through 70 uM and 40 uM cell strainers (Greiner Bio-one), where cells were counted, plated and expanded to second passage, then cryopreserved in freeze media (90% fetal bovine serum (FBS), 10% DMSO). For treatment, synoviocytes were thawed in 37°C water bath and recovered overnight in complete media. Cells were plated at 100,000 cells per well in a 24-well cell culture plate.

Synoviocytes were stimulated with IL-1β (20 ng/mL, R&D Systems) and TNF-α (20 ng/mL, Peprotech) and treated with activated MSC conditioned culture media (MSC CM) at a ratio of 1:3 MSC CM:complete media (Pezzanite L. et al., 2021). The stimulated and treated synoviocytes were cultured for 24 h, then washed with PBS, and cultured for an additional 24 h in complete media. At that time, synoviocyte supernatant was collected and frozen at −20°C for cytokine analysis (multiplex bead assay and ELISA immunoassay), and synoviocytes were collected in RLT lysis buffer and frozen at −20°C until RNA isolation was performed.

Chondrocytes were isolated from cartilage from the femur and caudal surface of the patella of the femoropatellar joint, as previously described (Pezzanite L. M. et al., 2021). Cartilage tissue samples were digested as described for synoviocytes overnight, passed through cell strainers, expanded in culture and cryopreserved for use at low passage prior to re-differentiation as previously described (Pezzanite L. et al., 2021). Chondrocytes were stimulated and treated as for synoviocytes above, and supernatant and chondrocytes in RLT lysis buffer were collected as above.

Macrophages were isolated from whole blood of one horse as previously described (Ma et al., 2014; Pezzanite et al., 2023). Monocyte-derived macrophages were stimulated with IL-1β (20 ng/mL) and TNF-α (20 ng/mL), and treated with activated MSC conditioned culture media at a ratio of 1:3 MSC CM:macrophage media. Controls included non-stimulated macrophages, and macrophages stimulated with IL-1β/TNF-α with no MSC treatment. The stimulated and treated macrophages were cultured for 24 h, then washed with PBS, and cultured for an additional 24 h in macrophage media. At that time, macrophage supernatant was collected and frozen at −20°C for cytokine analysis (multiplex bead assay and ELISA immunoassay), and macrophages were collected in RLT lysis buffer and frozen at −20°C until RNA isolation was performed.

### Cytokine and PGE-2 concentration determination

MSC and treated cell supernatants were analyzed for cytokine concentrations using a bead-based multiplex assay (Milliplex MAP Equine Cytokine/Chemokine Magnetic Beads Multiplex Assay, Millipore Sigma). The multiplex assay was used to quantify the concentrations of 23 equine cytokines. ELISA kits were used to measure PGE-2 (PGE-2 high sensitivity ELISA kit, Enzo Life Sciences), and TGF-β (Quantikine ELISA, R&D Systems, Inc.) concentrations.

### Transcriptomic analyses

Total RNA was isolated from activated MSCs and target cells and sent to Novogene Corporation Inc. for bulk RNA sequencing. Briefly, RNA was extracted from frozen samples in RLT lysis buffer using the RNeasy kit (Qiagen) according to manufacturer’s instructions. Total RNA sample quality and quantity was verified with Agilent 5400 Fragment Analyzer system (Agilent). RNA integrity number ranged from 7.3 to 9.7. Library was constructed with Abclonal Fast RNA-seq Lib Prep Kit V2 (ABclonal Technology). Samples were sequenced on NovaSeq X Plus, 150 bp paired end sequencing.

### Data analysis

Cytokine data was assessed for normality via Shapiro-Wilk tests, and visual assessment of diagnostic plots. The effect of activation of MSC supernatant treatment on cytokine production was evaluated by one-way ANOVA with *post hoc* Tukey’s adjustment for multiple comparisons (normal data) or Kruskal–Wallis test (non-normal data). Statistical analyses, graph analyses and graphical representations were performed using Prism Software v9.1.1. Statistical significance was established as p < 0.05.

RNAseq data was analyzed on Partek Flow v10 (Illumina, Inc. San Diego, California). Median reads for 33 samples were 47,709,688 pre alignment, sequences were trimmed for min Pred value of 20, adapters were trimmed with CUTADAPT v1.12 (Martin, 2011). Trimmed reads were aligned with STAR - 2.7.3a (Dobin et al., 2013) with reference genome Equab3.0. Aligned reads were counted with HTSeq v0.11.0 (Putri et al., 2022) with Ensembl gene annotation 108. Features were filtered for “protein coding” and lowest total coverage of 10 counts, DESeq2 was used for differential analysis (Love et al., 2014). Median ratio normalized counts were used for pathway analysis using GSEA v4.3.2. Gene sets used include Hallmarks, Reactome, WikiPathways, KEGG and Biocarta (UC San Diego).

## Results

### MSC activation with either TLR or STING stimulation induced differential gene expression compared to untreated MSC

**FIGURE 2:**
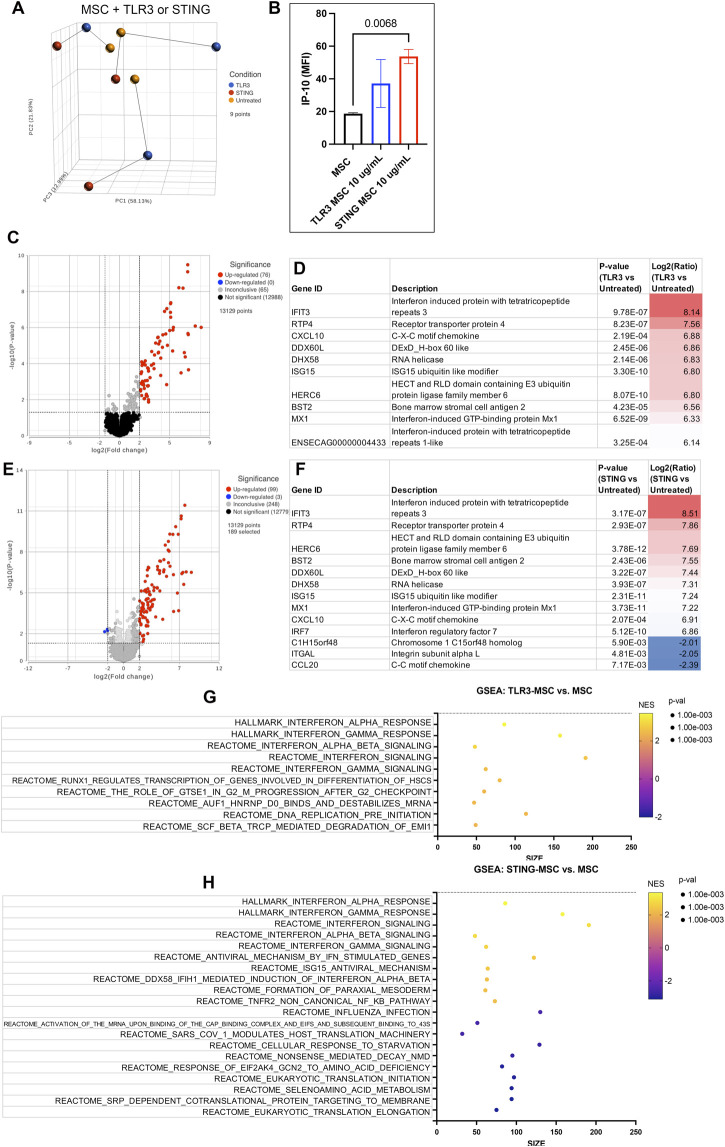
Transcriptome of MSC treated with innate immune stimulants. **(A)** Principal component analysis plot (PCA) of dimensionality reduced samples of n = 3 biological replicates of MSC treated with either TLR agonist (blue), STING agonist (red) and untreated (yellow). Lines show connecting biological replicates. **(B)** IP-10 cytokine secretion results of untreated MSC compared to either TLR- or STING-MSC-CM. Statistical analyses were performed using one way ANOVA with Tukey *post hoc* test. P value for significant comparison only (p<=0.05) shown on graph. **(C)** Volcano plot of differential gene expression analysis comparing protein coding genes in the TLR-MSC-CM to resting MSC. Significance defined as unadjusted p-value≤0.05. Fold Change (FC) ≥2Log_2_ or ≤ −2 Log_2._ Significantly upregulated genes shown in red, significantly downregulated genes shown in blue. **(D)** List of top 10 significantly upregulated genes in panel C comparison. **(E)** Volcano plot of differential gene expression comparing STING-MSC-CM to resting MSC. **(F)** list of significant genes from panel E, top 10 upregulated and all 3 of the significantly downregulated genes. **(G)** Gene set enrichment analysis (GSEA). Top 10 highest enrichment score (NES) upregulated pathways comparing treated TLR3 MSC to resting MSC. X-axis denotes the number of genes found in corresponding pathways (size). Enrichment score colored from purple (−2 downregulated) to yellow +2 enrichment score, upregulated. Size of dots show p-value for significance. All pathways shown filtered for unadjusted p-value of ≤0.05 and FDR adjusted p-value of 0.25. **(H)** GSEA of top 10 upregulated and top 10 downregulated pathway results comparing STING treated MSC to resting MSC.

Activation of bone marrow MSCs with either a TLR3 agonist (Poly I:C) or a STING pathway agonist (2′3′-cGAMP) altered the transcriptome significantly and increased secretion of chemokine IP-10 (Figure 2, Supplementary Document 1). TLR3 activated MSC showed significant upregulation of 76 protein coding genes in TLR3 activated cells and while there were 99 protein coding genes significantly upregulated in STING pathway activated MSC. Both treatments produced a strong upregulation of interferon genes such as IFIT and MX1. Both agonists upregulate pathways involving interferon signaling, as well as cell cycle and DNA response. In addition, STING agonist treatment also downregulated pathways related to protein translation and synthesis.

### Exposure of joint cells to TLR3 activated MSC reduced secretion of inflammatory cytokines

**FIGURE 3:**
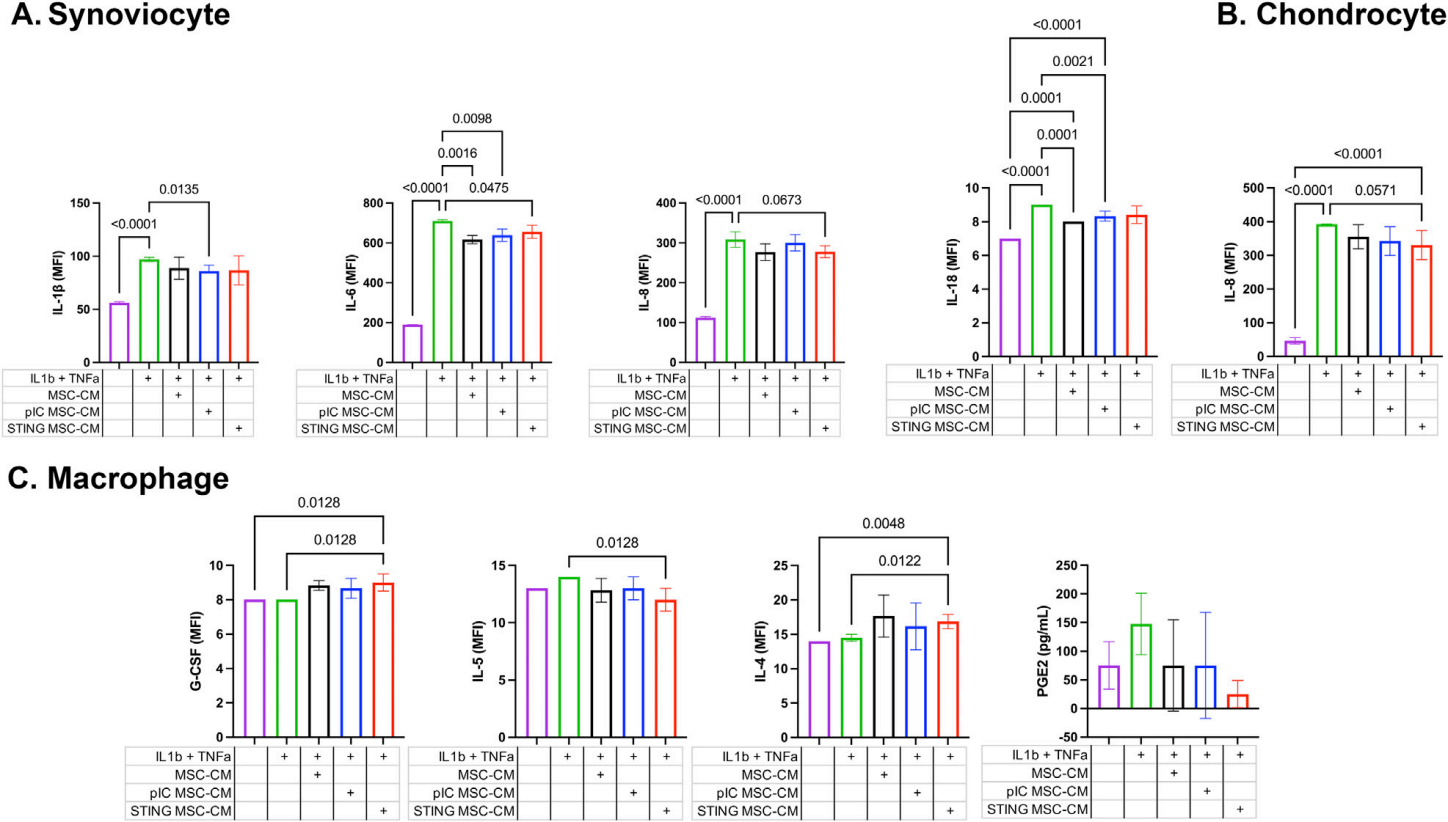
Cytokine and PGE-2 expression in cell culture supernatants of joint cells treated with activated MSC supernatants. Fluorescent bead-based multiplex assay and ELISA immunoassay were used to quantify concentrations of 23 analytes in cell culture supernatants of synoviocytes, chondrocytes, and macrophages treated with activated MSC conditioned media. Significant differences were noted in IL-1β, IL-6, and IL-8 levels for synoviocytes, IL-18 and IL-8 for chondrocytes, and G-CSF, IL-5 and IL-4 for macrophages. Results reported in MFI; * significance was assessed at P < 0.05. **(A)** Synoviocyte, **(B)** Chondrocyte, **(C)** Macrophage.

Three different target cell populations relevant to the joint environment (synoviocytes, chondrocytes and macrophages) were assessed for their cytokine responses after exposure to the secreted factors from activated MSCs following exposure to inflammatory stimuli TNF-α and IL-1β (Figure 3). TLR-MSC-CM reduced IL-1β and IL-6 production in synoviocytes, and reduced IL-18 secretion in chondrocytes. While STING treated MSCs also inhibited IL-6 in synoviocytes, they also reduced IL-8 in chondrocytes, and increased G-CSF and IL-4 production and decreased IL-5 levels in inflammatory macrophages.

### Secreted factors from activated MSC modulate immune and signaling pathways in synoviocytes

**FIGURE 4:**
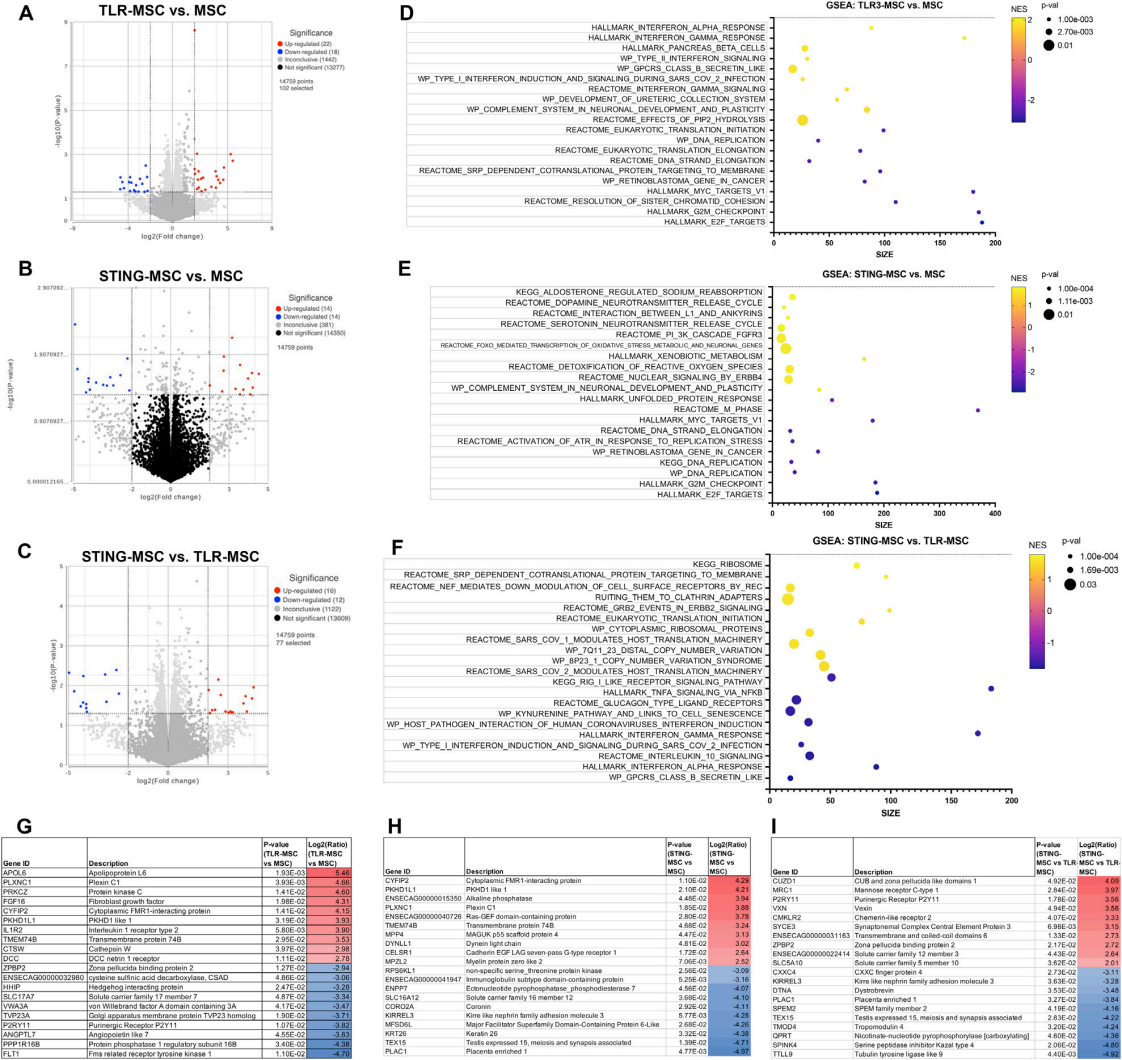
Transcriptome of synoviocytes treated with activated MSC supernatants. **(A)** RNAseq differential gene expression analysis results (protein coding genes) comparing n = 3 biological replicates of synoviocytes treated with TLR-MSC-CM compared to matched biological replicates of synoviocytes treated with resting MSC-CM. Figure shows volcano plot with significantly upregulated genes (p < =0.05, FC ≥ 2Log_2_) in red and significantly downregulated genes (p < =0.05, FC ≤ -2Log_2_) in blue. **(B)** Volcano plot of differential gene expression analysis (DESeq) comparing synoviocytes treated with STING-MSC-CM compared to resting MSC-CM. **(C)** Volcano plot of differential gene expression analysis comparing synoviocytes treated with STING-MSC-CM vs synoviocytes treated with TLR-MSC-CM. **(D–F)** top significant GSEA pathways of the above listed comparisons, using hallmarks, reactome, wiki pathways and KEGG. X-axis denotes the number of genes found in corresponding pathways (size). Normalized Enrichment score (NES) colored from purple (−2 downregulated) to yellow +2 enrichment score, upregulated. Size of dots show p-value for significance. All pathways shown filtered for unadjusted p-value of≤0.05 and FDR adjusted p-value of 0.25. **(G–I)** List of top 10 significantly upregulated and top 10 significantly downregulated genes in the above comparison.

Synoviocytes treated with TLR or STING-MSC CM showed minor changes in gene expression (Figure 4). TLR-MSC-CM treated synoviocytes had upregulation of immune pathways such as interferon, with genes such as APOL6, PRKCZ, IL1R2, IFIT3. STING-MSC-CM treated synoviocytes showed increased immune signatures, as well as a cellular signaling component and cytoskeletal function genes (CYFIP2, PKHD1L1, ACTL10). Direct comparison of STING-MSC-CM vs. TLR-MSC-CM treated synoviocytes revealed a total of 28 significant DEGs, the differences in transcriptome response are largely related to immune pathway upregulation in the TLR-MSC-CM treated synoviocytes whereas STING-MSC-CM treated synoviocytes had enrichment in pathways related to protein synthesis, signaling and metabolism.

### STING pathway activation resulted in enhanced immune response compared to TLR pathway activation in chondrocytes

**FIGURE 5:**
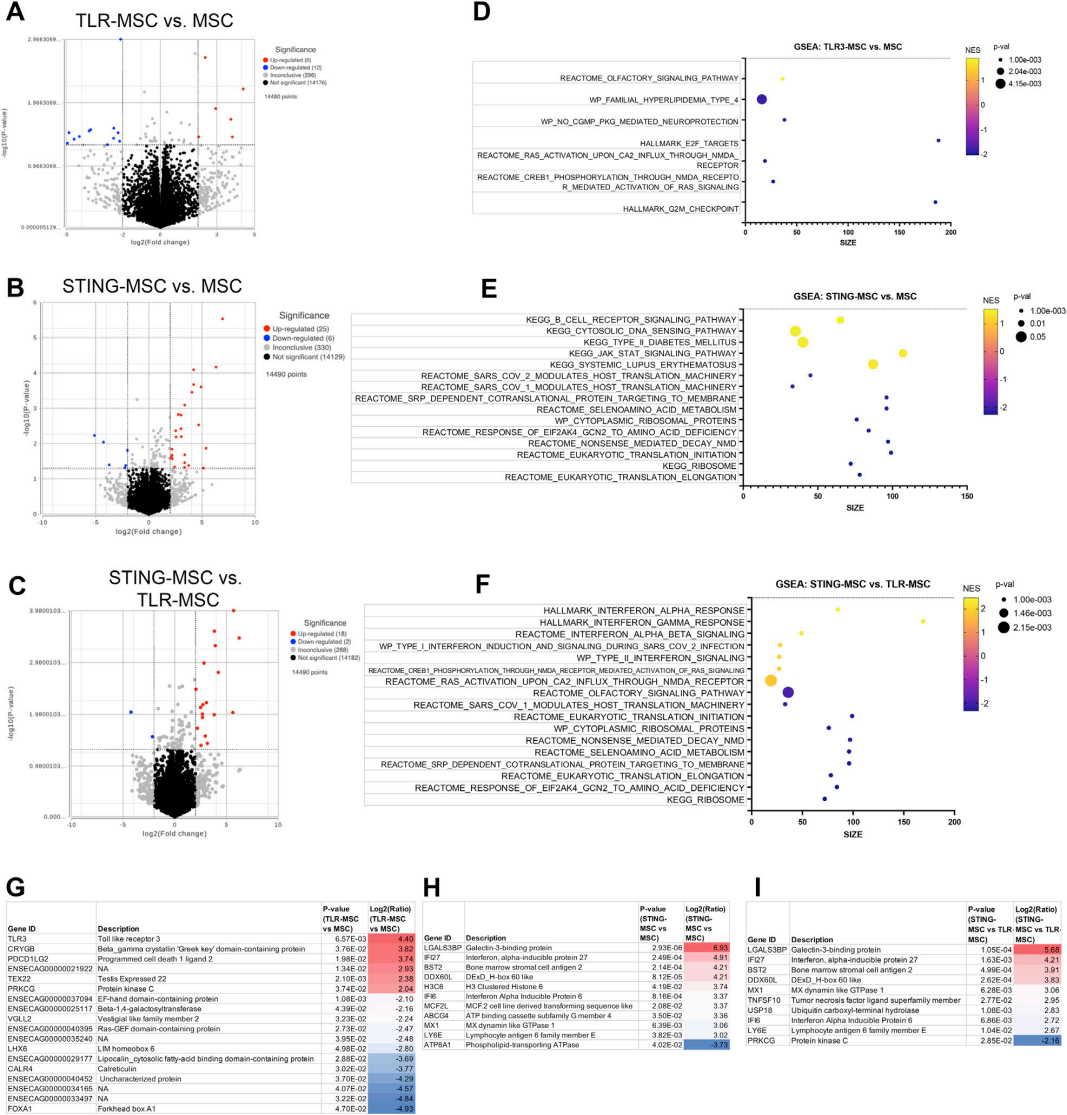
Transcriptome of chondrocytes treated with activated MSC supernatants. **(A)** RNAseq differential gene expression analysis results (protein coding genes) comparing n = 3 biological replicates of chondrocytes treated with TLR-MSC-CM compared to matched biological replicates of chondrocytes treated with resting MSC supernatant. Figure shows volcano plot with significantly upregulated genes (p < =0.05, FC ≥ 2Log_2_) in red and significantly downregulated genes (p < =0.05, FC ≤ -2Log_2_) in blue. **(B)** Volcano plot of differential gene expression analysis (DESeq) comparing chondrocytes treated with STING-MSC-CM vs chondrocytes treated with untreated MSC-CM. **(C)** Volcano plot of differential gene expression analysis comparing chondrocytes treated with STING-MSC-CM vs chondrocytes treated with TLR-MSC-CM. **(D–F)** top significant GSEA pathways of the above listed comparisons, using hallmarks, reactome, wiki pathways and KEGG. X-axis denotes the number of genes found in corresponding pathways (size). Normalized Enrichment score (NES) colored from purple (−2 downregulated) to yellow +2 enrichment score, upregulated. Size of dots show p-value for significance. All pathways shown filtered for unadjusted p-value of≤0.05 and FDR adjusted p-value of 0.25. **(G–I)** List of top 10 significantly upregulated and top 10 significantly downregulated genes in the above comparison.

Chondrocytes treated with TLR-MSC-CM revealed a small number (18 total) of significant DEGs (differentially expressed genes) (Figure 5). The top upregulated gene is TLR3, the target for poly I:C, followed by the crystalin gene which is likely part of the chondrocyte extracellular matrix (Bao et al., 2019). The most downregulated gene was FOXA1 associated with chondrocyte differentiation (Ionescu et al., 2012). Unlike synoviocytes, TLR-MSC-CM treated chondrocytes did not significantly upregulate any interferon or immune pathways. Treatment of chondrocytes with STING-MSC-CM resulted in additional upregulated genes and pathways including pro inflammatory genes Galectin 3, TLR and several interferons induced genes were upregulated and 6 significantly downregulated. The upregulated pathways include type 2 interferons alpha, gamma from multiple pathway databases and reactome pathways which include major categories such as translation and protein synthesis, cell cycle and DNA replication as well as signaling and response to cellular stimuli. Direct comparison of STING-MSC CM treatment vs TLR-MSC-CM treated chondrocytes showed 47 significant DEGs notably the immune pathways were much more overrepresented in STING-MSC-CM.

### Macrophages treated with stimulated MSC supernatant show increased metabolic gene expression

**FIGURE 6:**
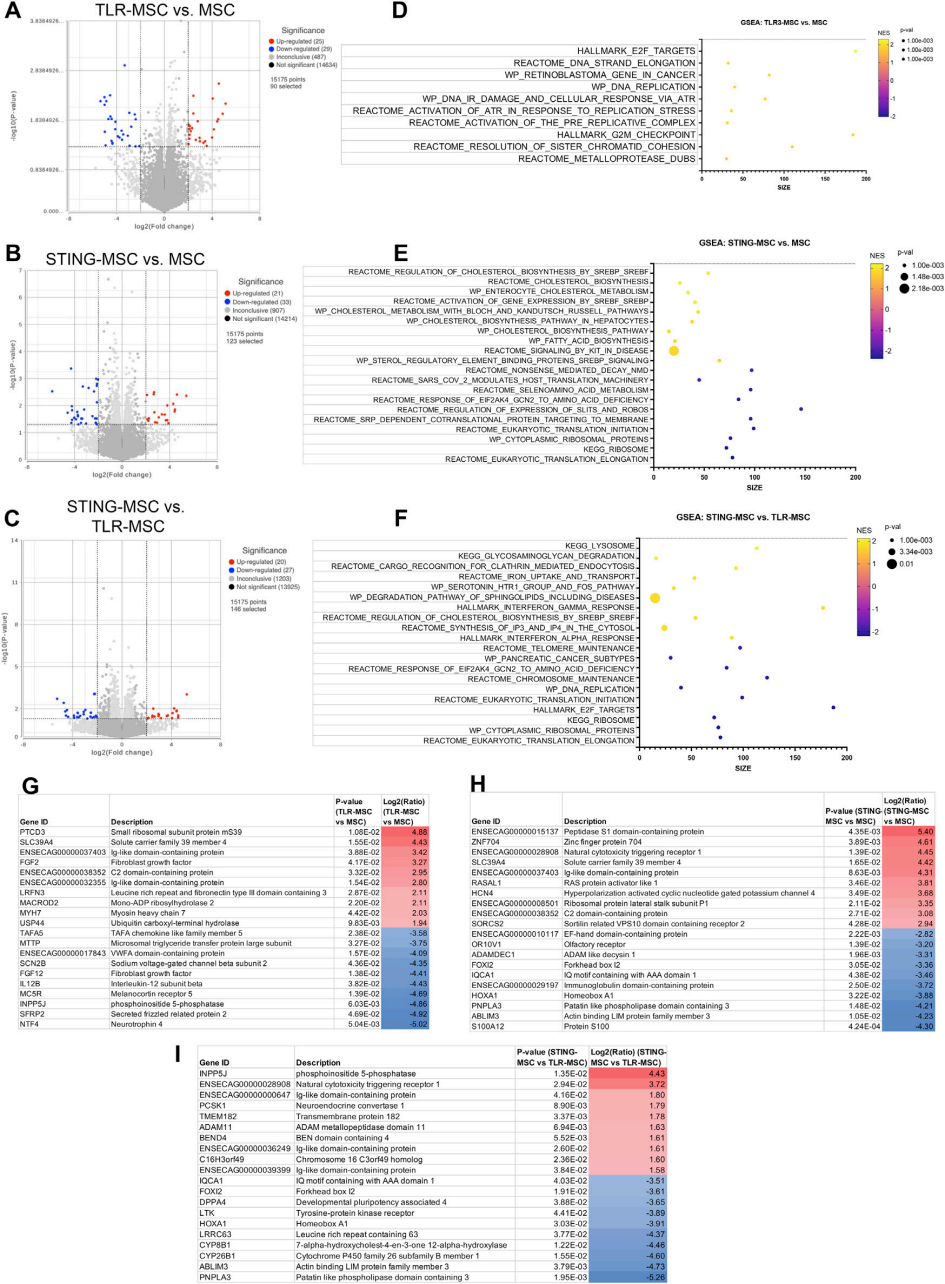
Transcriptome of macrophages treated with activated MSC supernatants. **(A)** RNAseq differential gene expression analysis results (protein coding genes) comparing n = 3 biological replicates of macrophages treated with TLR-MSC-CM compared to matched biological replicates of macrophages treated with untreated MSC supernatant. Figure shows volcano plot with significantly upregulated genes (p < =0.05, FC ≥ 2Log_2_) in red and significantly downregulated genes (p < =0.05, FC ≤ -2Log_2_) in blue. **(B)** Volcano plot of differential gene expression analysis (DESeq) comparing macrophages treated with STING-MSC-CM vs macrophages treated with untreated MSC-CM. **(C)** Volcano plot of differential gene expression analysis comparing macrophages treated with STING-MSC-CM vs macrophages treated with TLR-MSC-CM. **(D–F)** top significant GSEA pathways of the above listed comparisons, using hallmarks, reactome, wiki pathways and KEGG. X-axis denotes the number of genes found in corresponding pathways (size). Normalized Enrichment score (NES) colored from purple (−2 downregulated) to yellow +2 enrichment score, upregulated. Size of dots show p-value for significance. All pathways shown filtered for unadjusted p-value of≤0.05 and FDR adjusted p-value of 0.25. **(G–I)** List of top 10 significantly upregulated and top 10 significantly downregulated genes in the above comparison.

Macrophages treated with TLR-MSC-CM have significant transcriptomic changes compared to the other 2 joint cell types (Figure 6). These genes map to a diverse category of upregulated pathways such as those related to DNA Replication, Repair, and Cell Cycle. STING MSC-CM treated macrophages have a similar number of DEGs, but upregulate cholesterol metabolism, biosynthesis, and signaling, along with some other metabolic and disease-related pathways. Downregulated pathways are related to translation and ribosome function, and metabolic pathways. Although the direct comparison of STING-MSC supernatant treated vs TLR-MSC supernatant treated macrophages only showed a small number of significant DEGs (47 total), there were many pathways that were up or downregulated that were not seen in the above comparisons. Upregulated pathway can be broadly described in three categories including translation and ribosome function, disease and pathological conditions, as well as some reactome cellular responses and metabolic pathways.

### Interferon induced factors and complement cascade proteins regulated gene expression in joint cells

**FIGURE 7:**
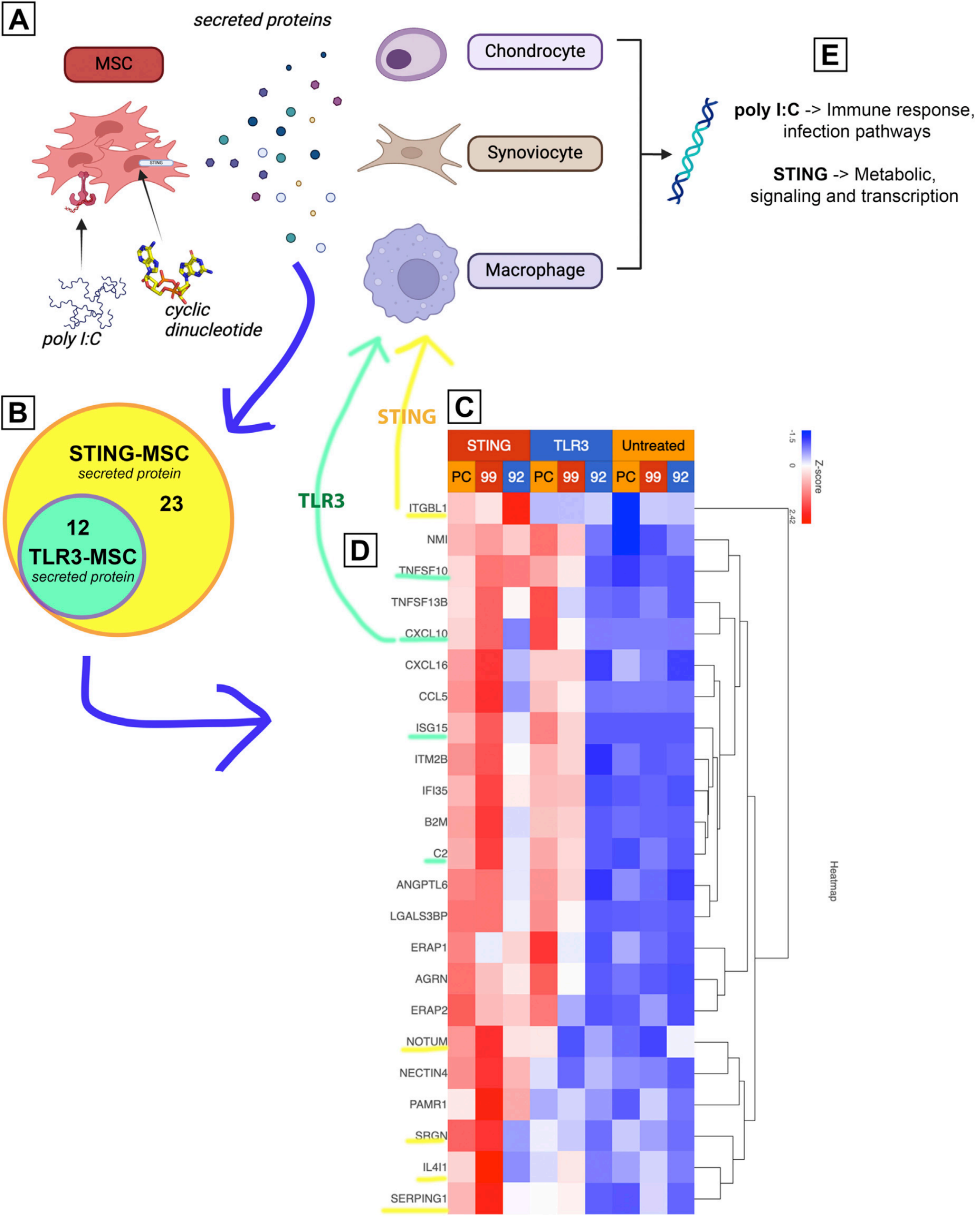
Proposed mechanism of increased treatment efficacy using stimulated MSCs based on joint cell type interaction with MSC secreted proteins. Figure shows diagram depicting experimental workflow. **(A)** shows MSCs treated with either TLR or STING agonist, then secreting different proteins. **(B)** upregulated protein coding genes (Figure 2) were matched to human protein atlas for prediction of secreted protein, which included 12 in the TLR treated MSCs and an additional 23 in the STING MSC shown in a Venn diagram. **(C)** Heat map of normalized expression values of 35 predicted secreted proteins (data from Figure 2) including 3 biological replicates (PC, 99, 92) as well as all treatment groups. Untreated in yellow, TLR3 in blue (top) and STING in red (top). **(D)** Yellow and green highlighted proteins were found to affect downstream pathways in 3 joint cell types (chondrocytes, synoviocytes and macrophages). **(E)** Key take aways from pathway matching.

Combining the transcriptome data from activated MSC and their effects on target cells, a prediction can be made on the potential mechanisms of how activated MSCs differ for OA treatment (Figure 7). First, gene expression results from non-activated and activated MSCs were matched to the human protein atlas (https://www.proteinatlas.org/humanproteome/tissue/secretome) to predict secreted proteins. TLR3 activation shows a predicted 12 upregulated secreted proteins, STING activation upregulated those same 12 proteins and included an additional 23 secreted proteins shown in the heatmap. Transcriptomic responses to activated MSC treatment from chondrocytes, synoviocytes and macrophages were then matched to these predicted secreted proteins. Among these proteins, in particular, TLR3 activated MSCs secreted TNFSF10, CXCL10, ISG15 and C2 (green highlight, 7C) were found upstream of several affected immune response and infection pathways in all 3 cell types. STING activation additionally increased proteins ITGBL1, NOTUM, SRGN, IL4L1, SERPING1, which influence downstream metabolic signaling and transcription pathways in the 3 joint cell types.

## Discussion

Priming or licensing MSCs with innate immune agonists can substantially alter the functionality of the cells, leading to more desirable properties when the cells are injected intra-articularly in patients with OA. In addition, MSC activation immediately prior to injection may reprogram the overall cell population to reduce cellular heterogeneity, which may be desirable from the standpoint of reducing MSC donor-to-donor variability (Krampera, 2011; Szabó et al., 2015; Cassano et al., 2018). The use of TLR3 agonists to license MSC has been reported by numerous groups in addition to ours, for application to suppression of inflammatory diseases such as multiple sclerosis. In our studies, we have primarily focused on TLR3 agonist activation of MSC to stimulate their anti-infective properties (Johnson et al., 2017; Pezzanite L. et al., 2021; 2022). However, we have recently shown that intra-articular injection of MSC activated with the TLR3 agonist poly I:C as well as the STING pathway agonist 2′,3-cGAMP significantly improved histologic and functional gait outcomes in a murine model of OA (Plaisance et al., 2025). Therefore, in the present study we sought to understand in greater detail potential mechanisms of interaction between activated MSC and their target cells in the joint, using an *in vitro* model system.

When examining transcriptomic responses of MSC themselves following activation, TLR3 activation responses were dominated by interferon responses, consistent with prior reports of MSC and tumor cells activated with these agonists. Following TLR3 activation, 76 genes were upregulated in MSC, with the top 10 representing interferon induced or related genes. Of the 12 genes representing secreted proteins, these notably included interferon stimulated gene 15 (ISG15) and agrin (AGRN), which have shown beneficial effects in chondrogenesis (Hwang et al., 2024) and cartilage formation (Eldridge et al., 2016). Mechanistically, ISG15 has been shown to increase USP18 (Basters et al., 2017), an IL-6 and IL-18 inhibitor (Jiang et al., 2021), and AGRN works to repair cartilage by attracting tissue progenitor cells and differentiation of articular chondrocytes through simultaneous activation of CREB and suppression of canonical Wnt signaling (Eldridge et al., 2020). Production of type I interferons by immune cells and sensory neurons has been described to elicit antinociceptive effects in murine models of neuropathic pain (Donnelly et al., 2021), with potential implications for pain mitigation in OA.

When examining the transcriptomic and cytokine response of MSC to 2′3′-cGAMP activation, an additional 23 genes were upregulated and an additional 3 genes were downregulated, compared to TLR3-activated cells. Of the top 10 upregulated genes, 9 were the same as in TLR3-MSC with IRF7, the “master regulator of type-1 interferon dependent-immune response s (Honda et al., 2005),” rising into the top 10. Gene expression pathway analysis demonstrated a similar increase in interferon dominated pathways in cells activated by STING. Moreover, there was also significant upregulation in ISG15 signaling and NF-kB pathway. Upregulated gene predicted to be secreted proteins exclusive to 2′3′-cGAMP activation show efficacy in reducing osteoarthritis or enhancing cartilage formation (ITGBL1, Notum, serpinG1). Integrin beta-like 1 (ITGBL1) inhibits integrin-ECM interactions and promotes chondrogenic differentiation (Song et al., 2018), and inhibits cartilage inflammation decreasing TNF-α, IL-1β, and IL-6 (Xie et al., 2023). Notum stimulates repair (González-Guede et al., 2024) of cartilage as a negative regulator of the Wnt pathway (Wang et al., 2019). SerpinG1, a serine protease inhibitor may also inhibit catabolism and inflammation of cartilage but has not been extensively studied (Wilkinson, 2021). Finally, STING activation induced increased IP-10 secretion from MSC, a chemokine known to be secreted in response to IFN-γ that further plays a role in activation and regulation of immune responses. These data provide further mechanistic insight to the effect of MSC priming with pattern recognition receptors, implicating induction of interferon responses as a primary pathway with relevance to in OA.

When examining transcriptomic and cytokine responses of synoviocytes to treatment with activated MSC-CM, TLR-MSC-CM treatment induced an increase in interferon response and downregulation of translation related pathways. Differentially expressed genes with known relevance to OA and cartilage homeostasis include protein kinase C (PKC), FGF16, and IL1r2. PKC has been implicated to have dual roles in OA, both inhibiting inflammation through TNF-α induced ICAM-1 expression (Phalitakul et al., 2011) while increasing MMP-1, -3, and -13 production in cartilage (Koskinen et al., 2011). FGF signaling has been associated with several functions, including cell differentiation, proliferation and tissue repair. While some FGFs have demonstrated an effect in OA and cartilage (both regenerative and degenerative), literature supporting a role for FGF16 specifically in the context of OA is limited. IL1-r2 suppresses the immune response to IL-1 reducing arthritis in mice (Shimizu et al., 2015). STING activation of MSC induced downregulation of similar pathways, while the upregulated pathways include increases in reactome to PI3K cascade, detoxification of ROS and nuclear signaling by ERB84. The increased plexin C1 is notable, as it ameliorates injury, inflammation, apoptosis and ECM degradation of chondrocytes exposed to IL-1β (Meng et al., 2023). Finally, non-activated MSC treatment reduced secretion of IL-6 from synoviocytes, an immunomodulatory cytokine with potential dual roles in OA. In OA, IL-6 is released from synovial fibroblasts and may contribute to OA through production of inflammatory mediators and MMP production through the JAK/STAT pathway (Guerne et al., 1989; Bondeson et al., 2010; Nguyen et al., 2017) and MAPK/PI3K cascade (Wiegertjes et al., 2020). TLR3 activated MSCs also reduced IL-18 in chondrocytes and IL-6 in synoviocytes, and reduced IL-1β, a key cytokine in OA that amplifies synovitis and cartilage degradation, secretion in synoviocytes. STING-MSC treatment further reduced expression of IL-8 from synoviocytes, indicating a potential anti-inflammatory role as IL-8 is known to attract neutrophils to regions of inflammation.

When assessing transcriptomic and cytokine responses of chondrocytes to treatment with activated MSC media, there was a significant reduction in pathways involved with molecules targeted by E2F and molecules involved with NMDA receptor. Upregulation of TLR3 and Protein Kinase C genes suggest an inflammatory response by the cells that is not reflected in their secretory profile. STING-MSC conditioned media induced an increase in the JAK-STAT, B cell receptor, and cytosolic DNA sensing pathways, and reduced expression of translation related pathways. Notable increased genes include IFI6, a negative regulator of innate immune responses (Villamayor et al., 2023), and MCF2L which is correlated to cartilage maintenance (Mitchell et al., 2013; Shepherd et al., 2015). STING-MSC also reduced expression of IL-8, which has been correlated to severity of OA through upregulation of MMP levels and chondrocyte hypertrophy.

When assessing transcriptomic and cytokine responses of macrophages to treatment with activated MSC media, TLR-MSC-CM treatment did not induce significant changes in cytokine secretion but did induce upregulation of DNA replication pathways and cell cycle pathways. Significant changes in individual genes show an upregulation of FGF2, an ambivalent mediator in OA depending on conditions in the joint (Li et al., 2024). STING-MSC treatment exerted significant effects in secretory changes, and changes in genetic expression with an upregulation in mainly cholesterol and fatty acid synthesis pathways. A downregulation in translational pathways also occurred. Significant changes in secretory cytokines of macrophages happened only when MSCs were activated with STING agonist, causing an increase in G-CSF and IL-4 while reducing secretion of IL-5. Macrophages, a key regulator in joint inflammation, in the synovial joint include synovial macrophages and infiltrating macrophages are classified as either M1, an inflammatory cytokine producing or destructive macrophage, or M2, an anti-inflammatory or reparative macrophage. Polarization states of these macrophages are essential for modulating the inflammatory state of the synovial joint. Granulocyte stimulating factor (G-CSF), shown to repair osteochondral defects (Okano et al., 2014) and damaged cartilage (Sasaki et al., 2017), decreases the M1/M2 ratio of macrophages (Wen et al., 2019). IL-4 is associated with M2 macrophages and inducing M2 polarization (Keegan et al., 2021) and is also chondroprotective in osteoarthritis (van Helvoort et al., 2022). IL-5 promotes eosinophilic inflammation (Dougan et al., 2019), suggesting its reduction beneficial in inflammatory diseases, but its implications are not fully characterized in the context of OA. Taken together these alterations in transcriptomic and cytokine responses with STING-MSC treatment of macrophages in particular support an immunomodulatory and overall anti-inflammatory role for STING induction in OA.

Limitations of this study include the *in vitro* nature of study design, short time course in culture, use of preclinical model tissues, and small donor horse sample size with some inter-individual variability noted between primary cells lines in differential gene expression and cytokine secretion. While inflammation is now an accepted hallmark of OA, our knowledge of the processes driving local and systemic inflammation resulting in progressive joint damage, and how this inflammation may be mitigated or modified by activated MSC therapy, are at present incomplete. In this context, IL-1β and TNF- α were used in this study as potent stimulators and mediators of inflammation; however, it is acknowledged that other potential inflammatory mediators such as calcium pyrophosphate crystals which are present in the joints of some individuals with OA may be investigated alternatively due to their clinical relevance, which was outside the scope of the current experiment. Given the preliminary preclinical *in vitro* nature of study design, there is potential for lack of translation of findings to the *in vivo* osteoarthritic environment, and it is acknowledged that the culture conditions described here could not replicate the spectrum of disease processes encompassed by OA nor be generalized to embody the complex, chronic pathologies that may progress over decades. The equine preclinical model was selected for its tissue availability and comparable disease prevalence, similarity in joint volume, cartilage thickness and articular cartilage loading forces to humans, making it a valuable preclinical model for high-throughput screening of biological therapies (McIlwraith et al., 2012); however, it is acknowledged that other clinically relevant preclinical model of OA exist, the pros and cons of which have been discussed elsewhere (McCoy, 2015). The tissue donors in this study included both male and female animals but the impact of sex on OA progression was not specifically evaluated and has not been previously investigated in the equine species as it has been reported in others such as humans as mice, to the authors’ knowledge. Further studies in large animal models are necessary to further define the optimal timing of intervention following joint injury and determine long term clinical therapeutic effect of whole cell therapy or administration of cell secreted factors (e.g., exosomes). Moreover, the studies performed here were performed using MSC conditioned media alone to primarily assess the paracrine effect induced by MSC licensing, to minimize confounding factors induced by cell-to-cell interactions, and to assess potential effect of treatment with secreted factors alone that may be purified (e.g., exosomes) for assessment in subsequent experiments. It is acknowledged that it is also possible that cell-to-cell contact between MSC and cells in the joint produces qualitatively different responses than exposure to secreted factors, though these studies were beyond the scope of the current report. These findings, interpreted in light of future application in OA, corroborate our recent findings indicating a beneficial effect of activated MSC therapy in murine OA models and supports further investigation of these therapeutic strategies in large animal models *in vivo*.

## Conclusion

These findings indicate that innate immune activated MSC can secrete factors that alter responses by chondrocytes, synoviocytes, and macrophages in often unpredictable ways. Our findings suggest that other secreted factors, including factors secreted as part of interferon pathway activation, are likely to be more important in the overall functional effects of activated MSC as intra-articular therapy for OA. MSC activation by the TLR3 and STING pathways induce few shared responses, and many unique responses, which suggests that closer examination of the shared pathways may provide new insights into common mechanisms of action for MSC activated by the two different innate immune pathways.

## Data Availability

The datasets presented in this study can be found in online repositories. Data have been deposited into the NCBI repository with accession number GSE277125.
