# Supplementary figures and images for: Innate immune pathway activation to modulate mesenchymal stromal cell (MSC) interactions with synovium and cartilage
Source: Front Bioeng Biotechnol. 2025 Aug 8;13:1605148. doi: 10.3389/fbioe.2025.1605148 (PMC12370743; doi:10.3389/fbioe.2025.1605148)

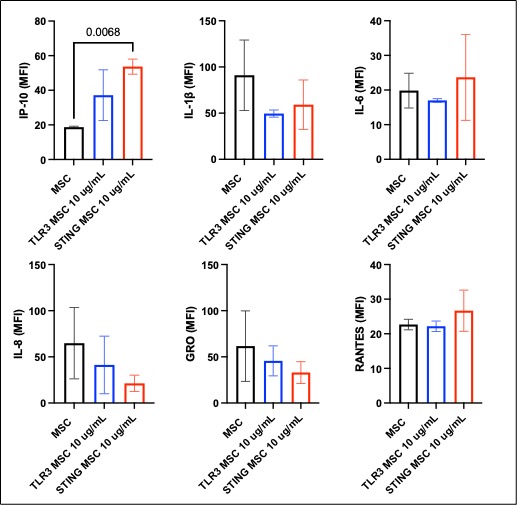

Supplement: Supplementary file 1 [file Image1.jpeg]
